# Compromised Photosynthetic Electron Flow and H_2_O_2_ Generation Correlate with Genotype-Specific Stomatal Dysfunctions during Resistance against Powdery Mildew in Oats

**DOI:** 10.3389/fpls.2016.01660

**Published:** 2016-11-08

**Authors:** Javier Sánchez-Martín, Gracia Montilla-Bascón, Luis A. J. Mur, Diego Rubiales, Elena Prats

**Affiliations:** ^1^Institute for Sustainable Agriculture, Consejo Superior de Investigaciones Científicas (CSIC)Córdoba, Spain; ^2^Institute of Biological, Environmental and Rural Sciences, Aberystwyth UniversityAberystwyth, UK

**Keywords:** *Avena sativa*, hypersensitive response, oat, penetration resistance, powdery mildew, resistance cost, stomatal dysfunctions, stomatal responses to pathogens

## Abstract

Stomatal dysfunction known as “locking” has been linked to the elicitation of a hypersensitive response (HR) following attack of fungal pathogens in cereals. We here assess how spatial and temporal patterns of different resistance mechanisms, such as HR and penetration resistance influence stomatal and photosynthetic parameters in oat (*Avena sativa*) and the possible involvement of hydrogen peroxide (H_2_O_2_) in the dysfunctions observed. Four oat cultivars with differential resistance responses (i.e., penetration resistance, early and late HR) to powdery mildew (*Blumeria graminis* f. sp. *avenae*, *Bga*) were used. Results demonstrated that stomatal dysfunctions were genotype but not response-type dependent since genotypes with similar resistance responses when assessed histologically showed very different locking patterns. Maximum quantum yield (Fv/Fm) of photosystem II were compromised in most *Bga*–oat interactions and photoinhibition increased. However, the extent of the photosynthetic alterations was not directly related to the extent of HR. H_2_O_2_ generation is triggered during the execution of resistance responses and can influence stomatal function. Artificially increasing H_2_O_2_ by exposing plants to increased light intensity further reduced Fv/Fm ratios and augmented the patterns of stomatal dysfunctions previously observed. The latter results suggest that the observed dysfunctions and hence a cost of resistance may be linked with oxidative stress occurring during defense induced photosynthetic disruption.

## Introduction

Powdery mildew is an important foliar disease of cultivated oat, *Avena sativa* L. caused by the biotrophic fungus *Blumeria graminis* (DC.) E.O. Speer f. sp. *avenae* Em. Marchal. This disease is common in humid temperate climates widespread in north west Europe and in south east United States of America causing yield losses up to 40% (Hsam et al., 2014). Although great progress has been made in limiting the effects of pathogen pressure, these are often made to the detriment of environment and human health through use of biocide agrochemicals. Perhaps the most sustainable approach to fight pathogens is through the better employment of resistant cultivars in breeding programs and in the field. In oats, resistance to powdery mildew is mainly determined by 7 major resistance (R) genes (*Pm*1 to *Pm*7; Hsam et al., 2014; Okori, 2015). These trigger a hypersensitive response (HR) when matches the corresponding avirulence gene in the pathogen, (Jones and Jones, 1979). In addition, so-called quantitative, non-HR based resistance, occurring mainly as adult plant resistance has also been observed (Montilla-Bascón et al., 2015). Although resistance have been described, care should be taken to avoid undesired effects such as rapid emergence of new virulent strains, unforeseen susceptibility to non-target diseases or pests, and particularly yield penalties through unexpected impacts on plant physiology and crop performance (Smedegaard-Petersen and Tolstrup, 1985) Given the current drive to improve food security through sustainable means it becomes imperative to establish the possible sources of resistance costs in order to circumvent them.

Cost of resistance is usually associated with the energetic and nutritional penalties linked to induction of defences (reviewed in Purrington, 2000; Heil and Baldwin, 2002; Brown, 2003; Burdon and Thrall, 2003). One of the first demonstrations of a resistance cost was in barley inoculated with an avirulent isolate of *Blumeria graminis* f. sp. *hordei* (hereafter *Bgh*) which exhibited a lower grain yield, smaller grains and less grain protein compared to uninoculated controls (Smedegaard-Petersen and Stølen, 1981). Nevertheless, until recently, this early evidence for a resistance cost had remarkably little impact on breeding for resistance (Swarbrick et al., 2006). However, renewed efforts in model plants are improving our understanding of the cost of disease resistance. For example, Tian et al. (2003) looked at the maintenance of alleles linked to resistance and susceptibility in a population of *Arabidopsis* lines and attributed a metabolic cost to the presence of one resistance (*R*) gene – *RPM1* (Tian et al., 2003). However, if there is a metabolic cost due to *RPM1*, it may be expected that there would be additive costs for all *R Arabidopsis* genes (estimated at more than 100) which would be evolutionarily prohibitive in the absence of persistent disease pressure (Brown, 2003; Burdon and Thrall, 2003). This point was recognized by Tian et al. (2003), who suggested additional factors responsible for the observed costs, possibly linked to the gratuitous induction of plant defense pathways in the absence of pathogens (Tian et al., 2003). The cost associated with the induction of defense responses and, in particular the cell death known as the HR, has also been the explanation of the low yield increase observed in the mixtures and multi-lines in which individual plants within a crop carry different *R* genes. However, a mechanistic understanding of the sources of these costs, other than vague suggestions of the energy “lost” in inducing the defense is lacking.

Our work in barley (Prats et al., 2006, 2010) and wheat (Prats et al., 2007a) showed that HR-mediated resistance provokes stomatal dysfunctions which could be an important component of the disease resistance cost. The HR cell death, provoked in barley by *Bgh*, was linked to a paralysation of stomata to “lock” open with severe physiological implications even when the attacked plants appear disease free (Prats et al., 2006, 2007a). A possible explanation of the stomatal dysfunction is the alteration of turgor balance of the epidermal stomata complex due to death of the nearby epidermal cells. This could cause the stomatal pore to open since opening depends on the balance between guard cell and subsidiary cell turgor. Whatever the mechanism, stomatal locking has clear implications on the plants ability to respond to drought stress (Prats et al., 2007a) and is in agreement with other authors that report a cost increase under stressful conditions (Heil et al., 2000; Heil, 2001; Heil and Baldwin, 2002). Another important question is the extent to which stomatal dysfunction occurs in other plant species or even genotypes within a species. If the extent of stomatal dysfunction varies amongst genotypes, this would ease the definition of its underlying cause(s) and would offer important opportunities for breeding.

In this work, we used a genotypically diverse series of oats (*Avena sativa*) cultivars (cvs) with different resistance responses to powdery mildew (*Blumeria graminis* f. sp. *avenae* (hereafter *Bga*) to (1) assess the extent to which stomatal dysfunction is directly related to the extent of HR and/or the different resistance responses (2) to explored the role of H_2_O_2_ and overall oxidative stress in the resistance-associated physiological dysfunctions (3) to identify oat cultivars with resistance response to powdery mildew displaying minimum physiological alterations upon pathogen attack.

## Materials and Methods

### Pathogen, Plants, and Inoculation

Freshly collected spores of *Bga* race 5, and *Bgh* CC1 were used. *Bga* and *Bgh* isolates were maintained on plants of susceptible oat cv. Selma and barley cv. Pallas, respectively in spore proof growth chambers. Plants were shaken to remove aging conidia 1 day before the inoculation.

Three oat cvs (Cory, Charming and Orblanche), previously identified as resistant to *Bga* race 5 and one susceptible (Selma) (Sanchez-Martin et al., 2011), were used for study of the oat-powdery mildew interaction. In addition the barley genotype P01 (Kølster et al., 1986) previously used in the characterisation of the stomatal dysfunctions in barley was added for comparison.

Plants were grown individually in 30 × 110 mm plastic centrifuge tubes (with two 5 mm drainage holes) filled with peat: sand (3:1). Tubes were stood in trays filled to a ∼50 mm depth in compost which was watered freely throughout. Plants were grown in a room with 20°C, 65% relative humidity and under 12 h dark/12 h light. Plants grew usually under a light intensity regime of 250 μmol m^-2^ s^-1^ photon flux density (hereafter referred to as low light, LL) but for the experiments of increased light intensity, after inoculation plants were subjected to a moderate light intensity regime of 450 μmol m^-2^ s^-1^ photon flux density supplied by high-output white fluorescent tubes (hereafter referred to as high light, HL).

For powdery mildew inoculation, the first fully expanded leaf (12 days seedlings), of the oat or barley plants was inoculated with *Bga* or *Bgh*, respectively, using a settling tower (Sanchez-Martin et al., 2011) to give about 30 conidia mm^-2^ (checked by counts made from glass slides laid adjacent to leaves).

### Microscopy

For histological studies, plants were maintained in the above mentioned growth chamber. Then at 36 h after inoculation (h.a.i) the central 30 mm leaf segment was excised and fixed on pads moistened with 3:1 ethanol:glacial acetic acid (v/v), and cleared with lactoglycerol (equal parts lactic acid, glycerol and water), as described before (Prats et al., 2007b) to avoid displacement of ungerminated conidia and loosely attached germlings. Four plants per genotype were analyzed at each fixation time under white and ultraviolet light incident fluorescent microscopy (330 nm excitation/380 nm emission) using a Leica DM LS phase contrast microscope (Leica Microsystems, Wetzlar, Germany; 40x objective).

Percentages of germlings hampered in the infection process before or at time of cell penetration (penetration resistance), percentage of germlings inducing early or late cell death and percentage of established colonies were determined from 100 germinated urediniospores per leaf segment. Death of attacked epidermal cells was recognized by whole-cell autofluorescence (Koga et al., 1990; Zeyen et al., 1995; Sanchez-Martin et al., 2011, 2012).

### Stomatal Conductance

Leaf water conductance (*g*_l_) was measured in ten plants per genotype with an AP4 cycling porometer that allows a non-destructive and rapid method for stomatal conductance measurement (Delta-T Devices Ltd, Cambridge, UK) as described in Prats et al. (2006). *g*_l_ is the sum of cuticular and stomatal conductance, but as cuticular conductance of oat is low (Bengtson et al., 1978), changes in *g*_l_ largely reflect changes in stomatal aperture. Stomatal conductance was measured on the center of the adaxial surface of leaf laminae (covering an area of 17.5 mm × 2.5 mm), of fully expanded second leaves twice a day, 3 h after the onset of the light period and 2 h before the end of the dark period. In light, a single measurement took <20 s, and in darkness slightly longer as the g_1_ was lower. Consequently, 10 plants were measured under 5 min. In each experiment, sets of 10 healthy and 10 inoculated plants of the chosen genotypes were measured, each set being held in adjacent trays on the growth room bench. The porometer was wiped clean after measuring inoculated leaves to avoid transferring the pathogen. Measurements in the powdery mildew highly susceptible cv. Selma were stopped earlier than in the other cvs in order to avoid experimental interference with sporulation.

To better visualize differences between treatments and genotypes the area under the conductance progress curve (AUCPC) of treated plants with respect to the control curve of non-stressed plants was calculated using the following formula

$$AUCPC=\overset{i=1}{\underset{k}{\Sigma }}1/2\left[\left(S_{i}+S_{i+1})(t_{i+1}-t_{i}\right)\right]$$

where S_i_ is the conductance at assessment date i, t_i_ is the number of days after the first observation on assessment date i and k is the number of successive observations.

### Chlorophyll Fluorescence Analysis

Fluorescence quenching analyses were measured using modulated fluorescence on second leaves of dark-adapted plants with a PAM 2100 Fluorometer (PAM-2000; Walz, Effeltrich, Germany). Measurements were made in four different replications according to (Sánchez-Martín et al., 2015).

The Fo was determined after dark adaptation (at least 30 min) with a pulsed low red measuring light (ML) (0.1 μmol photons m^-2^ s^-1^). Then, a 1-s saturating light pulse (∼6.000 μmol photons m^-2^ s^-1^] of white light was applied to measure the maximal fluorescence (Fm) value. When fluorescence returned again to the F_o_ level, plants were illuminated by a non-saturating continuous red “AL” (655nm) for 5 min to drive photosynthesis and gives F’ values. During the AL induced fluorescence kinetics, saturating pulses were applied with 20-s intervals in order to keep track of the fluorescence parameters Fm’ (the maximum fluorescence level in the light-adapted state). After several minutes under AL, and when the F value remained low and constant, AL was turned off and a far-red radiation (735 nm) that excited preferentially PS I was switched on during 4 s to determine the value of Fo′. During the induction kinetic induced by AL the ML is automatically switched to a higher frequency of 100 kHz in order to achieve a better signal to noise ratio and time resolution. In ML, while measuring F_0_, the frequency of 100 k Hz is replaced by 1.6 k Hz to avoid any chlorophyll fluorescence induction kinetics. Based on these basic parameters obtained, other parameters with significant physiological relevance such as i) Maximum Quantum Yield of PSII photochemistry (F_v_/F_m_) and ii) PSII operating efficiency (F′_q_/F′_m_ also termed ΔF/F_m_‘) were derived.

#### Dark Relaxation Measurements

Dark relaxation kinetics were used to determinate the q_I_ caused by photoinhibition of PSII units, a major constituent of the non-photochemical quench. Following turning off of the AL during the induction kinetics, a saturation pulse was applied within the first minute of darkness and then at 5 and 20 min. ML remained switched on throughout the dark relaxation measurements of q_N_. Then Fm‘1, Fm‘5 and Fm‘ 20 were obtained and used to determinate _N_F values (=Fm-Fm′) and hence q_N_ (_N_F/F_v_). The q_I_, was calculated as _N_F20/F_v._ (Lichtenthaler et al., 2005).

### H_2_O_2_ Measurement

H_2_O_2_ content was measured in five plants per genotype according to Wei et al. (2015) with some modifications. Approximately 100 mg of fresh weight was homogenized in 1 mL 0.1% trichloroacetic acid (TCA) in an ice bath. The homogenate was centrifuged at 12000 *g* for 20 min at 4°C. Then, the reaction mixture consisting of 75 μL of supernatant, 75 μL of 10 mM potassium phosphate buffer (pH 7.0) and 150 μL of KI was added to a microtiter well. The absorbance was measured at 390 nm 10 min later and was stable at least 30 min afterward. A calibration curve was performed with H_2_O_2_ standards at different concentrations in a similar way.

### Cell Membrane Stability

Cell membrane stability (CMS) was measured in five plants per accession according to Sánchez-Martín et al. (2015). Samples collected were washed three times in deionized water to remove electrolytes adhered on the surface. The samples were then inserted into a capped vial (20 mL) containing 10 mL of deionized water and incubated in the dark for 24 h at room temperature. The conductivity was measured with a conductivity meter (CMD 510, WPA, UK). After the first measurement, the vials were autoclaved for 15 min to kill the leaf tissue and release the remaining electrolytes. After cooling, the second conductivity reading was taken. These two measurements were carried out individually for all the samples from both the control and stress treatments. The control gave a measure of leakage solely due to the cutting and incubation of leaf disks. The conductance of the stressed sample was a measure of electrolyte leakage due to water stress, in addition to damage caused by cutting and incubation, and was assumed to be proportional to the degree of injury to the membranes. CMS was calculated as the reciprocal of cell-membrane injury after Blum and Ebercon, 1981:

CMS%=[(1−(T1/T2))/(1−(C1/C2))]×100,

where T and C refer to the treated and control samples, respectively; the subscripts 1 and 2 refer to the initial and final conductance readings, respectively.

### Statistical Analysis

All experiments were performed according to completely randomized designs. For ease of understanding, means of raw percentage data are presented in tables and figures. However, for statistical analysis, data recorded as percentages were transformed to arcsine square roots (transformed value = 180/II × arcsine [√(%/100)]) to normalize data and stabilize variances throughout the data range. Data were subjected to analysis of variance (ANOVA) using SPSS software for comparison of treatments and analysis of interactions between factors, after which residual plots were inspected to confirm data conformed to normality. Significance of differences between means was determined by contrast analysis (Scheffe’s). Pearson correlations were calculated to detect statistical correlations between traits measurements. In addition, least significant difference (LSD) values were added to tables for comparison.

## Results

### Microscopic Response of Oat Cultivars to *Bga*

To further test the correlation between the execution of different resistance responses and stomatal lock-up (Prats et al., 2007a, 2010), we evaluated the resistance responses and also the timing of HR development following *Bga* inoculation in several oat cultivars with different genetic backgrounds (**Table 1**).

**Table 1:** Microscopic assessment of fungal development and leaf epidermal cell responses in oat, Charming, Cory, Orblanche and Selma and barley, P01, plants inoculated with appropriate powdery mildew ff.spp.

Cultivar/fungus	Penetration resistance	Early HR	Late HR	Total HR	Colony
Charming/*Bga*	45.5 + 1.4	28.3 + 1.7	18.3 + 1.3	48.8 + 2.3	7.8 + 2.8
Cory/*Bga*	49.2 + 1.9	30.0 + 1.6	16.3 + 0.9	46.3 + 1.7	4.5 + 1.0
Orblanche/*Bga*	48.8 + 3.3	25.0 + 2.0	21.3 + 0.9	46.3 + 2.1	5.0 + 1.8
Selma/*Bga*	32.3 + 3.2	2.3 + 0.9	0.0 + 0.0	2.3 + 0.9	65.5 + 3.0
Barley P01/*Bgh*	49.3 + 2.1	42.0 + 2.3	4.0 + 2.4	46.0 + 3.0	4.8 + 1.0
l.s.d.^∗^	7.5	5.2	4	6.4	6.4

*Plants were grown under a 12 h dark/12 h light cycles and a light regime of 250 μmol m^-2^s^-1^. ^∗^Least significance differences (P < 0.05) for statistical comparisons*.

As expected, Selma was highly susceptible to *Bga* with more than 65% of attack sites forming colonies with no sign of visible HR. The remaining sites exhibited penetration resistance due to papilla formation. All other tested oat cvs were significantly more resistant than the susceptible Selma (**Table 1**). Cory, Charming and Orblanche showed similar levels of HR to *Bga*, being observed at nearly 50% of attack sites, with most of the remaining exhibiting penetration resistance (∼45%). These proportions were similar to that elicited by *Bgh* in the barley cv. P01 which was included to allow comparison with our previously published results (Prats et al., 2006, 2007a). However, whereas in P01 most of the HR developed very rapidly; before any haustoria could be seen in the epidermal cells, Charming, Cory and Orblanche, exhibited a higher and similar proportion of late cell death.

### Physiological Changes in Oat cvs. Following Challenge with *Bga*

#### Stomatal Conductance (g_l_)

**FIGURE 1:**
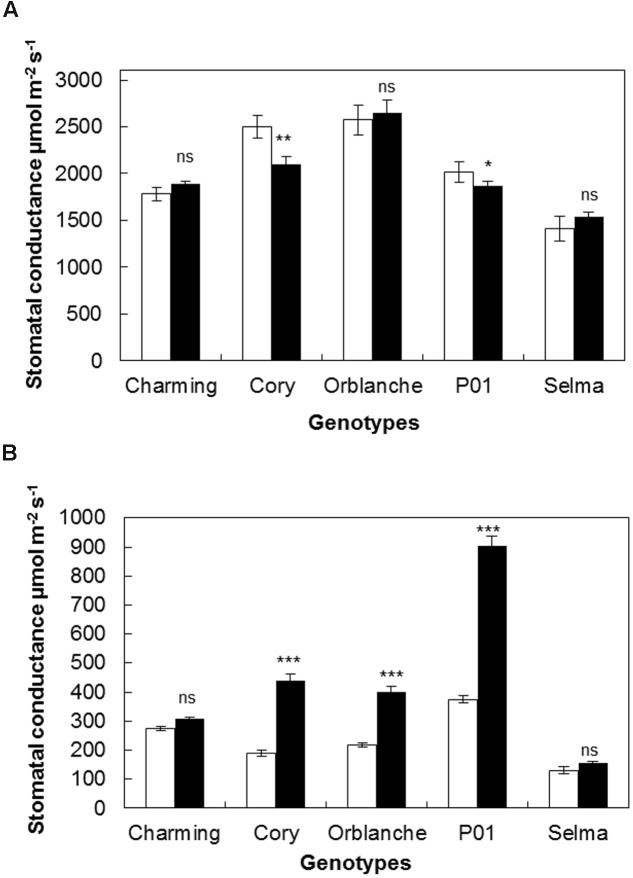
**Stomatal responses of oat and barley plants to powdery mildew attack.** Area Under the Conductance Progress Curve (AUCPC) corresponding to a time course of **(A)** daytime and **(B)** night-time leaf water conductance (g1) of healthy controls, (white bars) Charming, Cory, Orblanche, and P01 leaves and inoculated plants (black bars). Plants were grown under a 12 h dark/12 h light cycles under a light regime of 250 μmol m^-2^ s^-1^. Bars represent the mean of 10 biological replicates ± standard errors. ^∗^, ^∗∗^, and ^∗∗∗^ indicate significant differences at *P* < 0.05, *P* < 0.01, and *P* < 0.001 respectively; ns indicates non-significant differences.

Having characterized the responses of our panel of oat genotypes to *Bga*, we next investigated the stomatal responses to attempt pathogen attack. To more clearly display effects on stomatal conductance the results are given using AUCPC values (**Figures 1A,B**) but complete data along the monitored time course can be accessed in Supplementary Figure S1. During the day, in control healthy oat plants, stomatal conductance ranged between 300 and 500 μmol m^-2^s^-1^. Following inoculation, a significant reduction on stomatal conductance (*P* < 0.001) was observed in barley P01; confirming previous results. Of all the oat cultivars screened, only Cory exhibited a reduction that was similar to that observed in P01; with ∼15% reduction in the AUCPC values on inoculated plants compared to the non-inoculated control (*P* < 0.001, **Figure 1A**). Oat cvs. Charming and Orblanche and the susceptible cv. Selma exhibited no significant reduction in stomatal conductance during daytime. Measurements on Selma were stopped from 5 days after inoculation (d.a.i) as fungal sporulation could influence the measurements of *g*_l_ (**Figure 1A**).

Our previous work has suggested that stomatal locking was best seen during dark periods as increased conductance (Prats et al., 2006). During night-time (**Figure 1B**), most cvs showed increased conductance following powdery mildew inoculation. Thus, Cory, and Orblanche showed increased stomatal conductance from 2 d.a.i. (*P* < 0.001) (Supplementary Figure S1) with overall increases of ∼32% and ∼83% respectively in the AUCPC curves (**Figure 1B**). These increases were nevertheless far below the ∼142% increase in stomatal conductance observed in the barley P01 (*P* < 0.001, **Figure 1B**). In the case of Charming, there were no significant differences in stomatal conductance in control and inoculated plants during the experimental time course of 8 days (**Figure 1B**). No increases in the AUCPC of night-time stomatal *g*_l_ were observed in the susceptible Selma, albeit it started to show a significant higher night-time *g*_l_ from 5 d.a.i. (Supplementary Figure S1B). Measurements on Selma were stopped from this time point since fungal sporulation could influence *g*_l_ measurements. Then from the resistant oat cultivars tested only cv. Charming showed no effect of inoculation on diurnal or nocturnal conductance.

#### Chlorophyll Fluorescence

**FIGURE 2:**
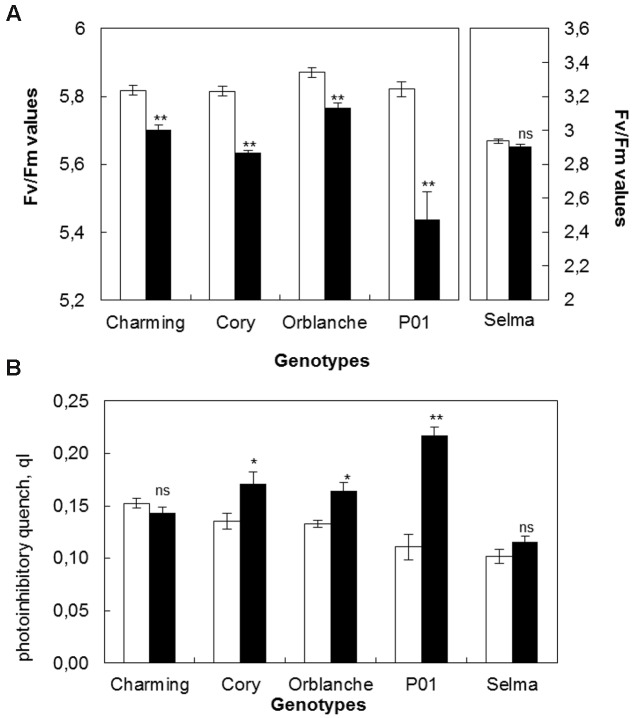
**Photosynthetic responses of oat and barley plants to powdery mildew attack. (A)** Area Under the Progress Curve (AUPC) corresponding to a time course of Maximum Quantum Yield (Fv/Fm) measurements and **(B)** qI of healthy (white bars) Charming, Cory, Orblanche, and P01 leaves and inoculated plants (black bars). Plants were grown under a 12 h dark/12 h light cycles under a light regime of 250 μmol m^-2^ s^-1^. Bars represent the mean of 5 biological replicates ± standard errors. ^∗^ and ^∗∗^ indicate significant differences at *P* < 0.05 and *P* < 0.01, respectively; ns indicates non-significant differences.

To relate pathogen impacts on stomata with overall plant physiology effects, the status of the photosynthetic electron transport was estimated through chlorophyll fluorescence parameters (**Figures 2A,B**). Similarly than for stomatal data, the results are given using the values corresponding to the area under the progress curve of the maximum quantum efficiency (Fv/Fm) of the complete time course. *Bga* inoculation significantly reduced Fv/Fm ratios in all oat cvs (**Figure 2A**). The highest reduction in Fv/Fm was observed in Cory while Charming and Orblanche exhibited a slightly lower reduction. However, overall, the highest reduction was observed in the barley cv. P01 challenged with *Bgh* with a reduction up to more than 50% (**Figure 2A**). In all cases, the highest reduction in Fv/Fm was observed around 7–8 d.a.i.

Dark relaxation measurements on inoculated oat and barley leaves showed that photoinhibition significantly increased in Cory, Orblanche and also in the barley P01, being this increase of more than 50% in P01 (**Figure 2B**). From the resistant cultivars assessed only Charming showed no increased photoinhibition following pathogen attack. Overall, and as happened with stomatal dysfunction, Charming cv. showed the smallest alteration in chlorophyll fluorescence parameters whereas Cory showed the highest alteration, similar or slightly lower than in the barley genotype P01.

### Changes in H_2_O_2_ Content and Associated Cell Damages Following Pathogen Inoculation

**FIGURE 3:**
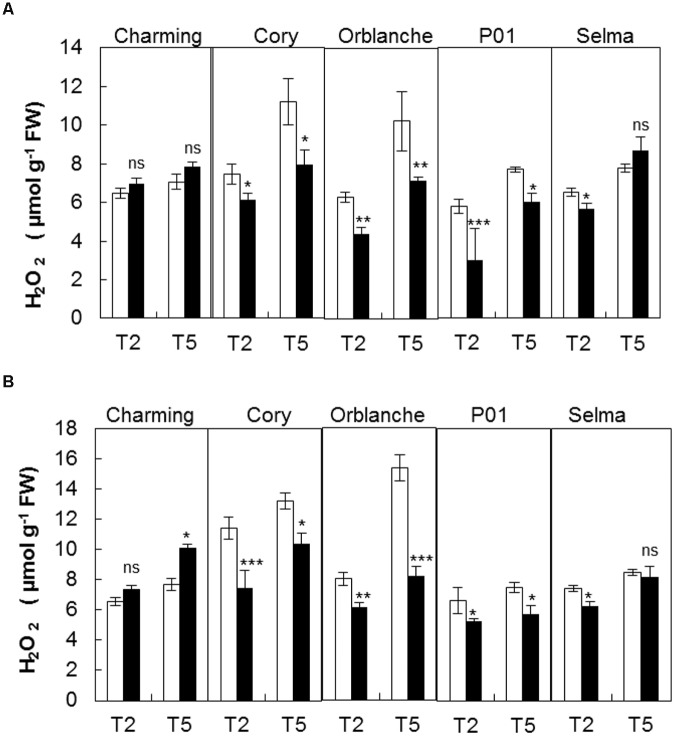
**Hydrogen peroxide production in oat and barley plants in response to powdery mildew attack.** Hydrogen peroxide was measured in control (white bars) and *Blumeria graminis* f. sp. *avenae* inoculated Charming, Cory, Orblanche, and P01 leaves (black bars) at 2 and 5 days after inoculation (T2 and T5). Plants were grown under a 12 h dark/12 h light cycles and a light regime of 250 μmol m^-2^s^-1^ **(A)** throughout the experiment or **(B)** transferred to a higher light intensity regime of 450 μmol m^-2^s^-1^ after inoculation. Bars represent the mean of 5 biological replicates ± standard errors. ^∗^, ^∗∗^, and ^∗∗∗^ indicate significant differences at *P* < 0.05, *P* < 0.01, and *P* < 0.001 respectively; ns indicates non-significant differences.

It is widely known that a transient oxidative burst is generated during the execution of the penetration resistance and HR (i.e., Lamb and Dixon, 1997, Piffanelli et al., 2004). In addition H_2_O_2_ is required for the complex signaling pathway that orchestrates stomatal movements (Pei et al., 2000). We therefore explored whether different H_2_O_2_ content in the resistant genotypes following inoculation could be related to the different stomatal and photosynthetic dysfunctions observed. Interestingly, most cultivars exhibited reduced H_2_O_2_ content 2 days after pathogen attack, albeit significant interactions between cultivars and inoculation treatment were observed. Thus, resistant cv. Charming showed no significant differences in H_2_O_2_ content whereas Cory, Orblanche and the resistant barley P01 showed decreases of approximately 25–30% in H_2_O_2_ content (**Figure 3A**). In some cvs this could reflect a diversion of reductants (e.g., NADPH) from a generalized whole tissue pattern of H_2_O_2_ to a more focused infection site specific oxidative burst. In this context, infection linked H_2_O_2_ content at 5 d.a.i. was higher than at 2 d.a.i. with no significant interaction between time and cultivars.

To investigate the link between general and localized oxidant/antioxidant metabolism on stomatal locking we explored increasing overall oxidative stress through slightly increase of the light intensity up to 450 μmol m^-2^ s^-1^. Fungal development and leaf epidermal cell responses under this light regime were assessed to check for any possible change of the resistance responses (**Table 2**). Overall, mildew development and plant cell responses to the fungi under the moderate high light intensity did not differ from that observed under the normal regime (Prats et al., 2007a, 2010). However, there were slight changes in penetration resistance.

**Table 2:** Microscopic assessment of fungal development and leaf epidermal cell responses in oat, Charming, Cory, Orblanche and Selma and barley, P01, plants inoculated with appropriate powdery mildew ff.spp.

Cultivar/fungus	Penetration resistance	Early HR	Late HR	Total HR	Colony
Charming/*Bga*	37.6 + 6.7	24.0 + 0.8	30.0 + 4.1	54.0 + 4.1	8.3 + 3.1
Cory/*Bga*	39.5 + 5.7	33.3 + 4.4	14.3 + 2.1	47.5 + 5.0	13 + 2.5
Orblanche/*Bga*	41.3 + 3.4	22.8 + 2.3	25.5 + 1.7	55.8 + 2.1	10.5 + 1.3
Selma/*Bga*	31.5 + 2.5	0.0 + 0.0	0.0 + 0.0	0.0 + 0.0	68.5 + 2.5
Barley P01/*Bgh*	58.3 + 2.9	36 + 3.8	0.0 + 0.0	39 + 1.5	4.0 + 0.8
l.s.d.^∗^	15.4	8.0	7.5	9.5	8.9

*Plants were grown under a 12 h dark/12 h light cycles and a light regime of 250 μmol m^-2^s^-1^ and transferred to a higher light intensity regime of 450 μmol m^-2^s^-1^ after inoculation. ^∗^Least significance differences (P < 0.05) for statistical comparisons*.

**FIGURE 4:**
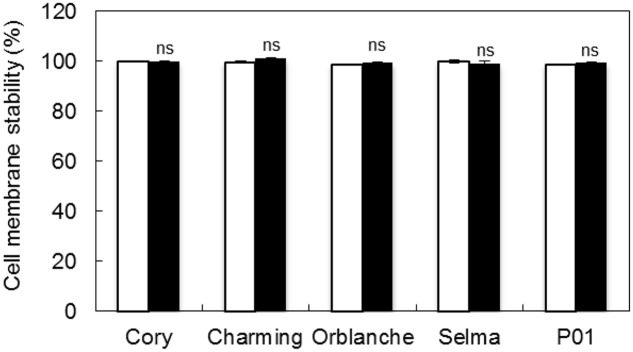
**Cell membrane stability (CMS) of oat and barley plants in response to powdery mildew attack.** CMS measured in inoculated Charming, Cory, Orblanche, P01 and Selma leaves with *Blumeria graminis* f. sp. *avenae* is expressed respect to non-inoculated controls plants. Bars represent measurements under two different light regimes, normal light intensity (250 μmol m^-2^ s^-1^, white) and moderate high light intensity (450 μmol m^-2^ s^-1^, black). Data are mean of 5 biological replicates ± standard error.

The increase of light intensity increased also significantly (*P <* 0.001) the overall H_2_O_2_ content but this was again reduced (*P <* 0.001) in response to pathogen attack in resistant cultivars, with the exception of Charming (**Figure 3B**). The susceptible cv. Selma did not exhibit the clear response observed in the resistant cultivars. There were interactions between all factors indicating that the different genotypes responded in a different manner to the light and inoculation treatment. However, overall, under the moderate high light regime we observed a slightly higher reduction of the H_2_O_2_ content in inoculated respect to control plants (**Figure 3B**). Assessment of CMS showed no cell damage consequence of oxidative stress under any of the light regimes used and after pathogen attack (**Figure 4**).

### Effect of Increased Light Intensity on Stomatal Responses and Photosynthetic Electron Flow

Next, in order to determine whether the changes in the oxidant/antioxidant metabolism could influence further the stomatal and the photosynthetic dysfunctions previously observed in the resistant genotypes, we assessed the effect of the light-induced changes on stomatal conductance and chlorophyll fluorescence parameters.

**FIGURE 5:**
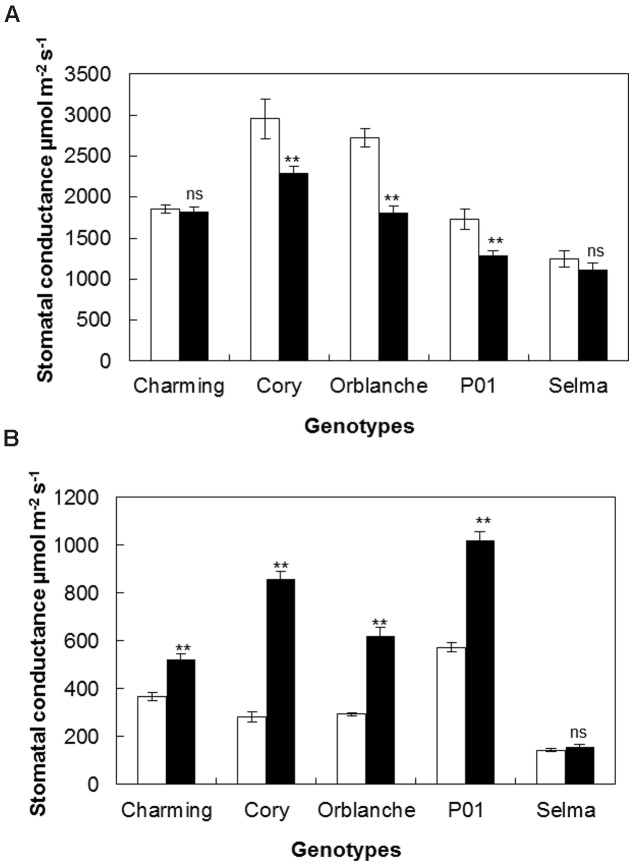
**Stomatal responses of oat and barley plants to powdery mildew attack and increased light intensity.** Area Under the Conductance Progress Curve (AUCPC) corresponding to a time course of **(A)** daytime and **(B)** night-time leaf water conductance (g1) of healthy (white bars) Charming, Cory, Orblanche, and P01 leaves and inoculated plants (black bars). Plants were grown under a 12 h dark/12 h light cycles and a light regime of 250 μmol m^-2^s^-1^ and then transferred to a higher light intensity regime of 450 μmol m^-2^s^-1^ after inoculation. Bars represent the mean of 10 biological replicates ± standard errors. ^∗∗^indicate significant differences at *P* < 0.01, respectively; ns indicates non-significant differences.

Following light intensity increase at time of inoculation, we observed a strong interaction regarding the responses of the different genotypes to the increased light and pathogen attack (*P* < 0.001). Thus, whereas Charming and Selma still did not show any differences in daytime conductance following inoculation, Cory, Orblanche and P01, showed higher decreases in diurnal g_1_ after pathogen attack than those observed under the normal light regime (**Figure 5A**). A similar effect was observed regarding night-time g_1_. Thus, there was a significantly (*P* < 0.001) higher stomatal lock-up in all inoculated resistant cvs under the increased light intensity compared with the normal intensity (**Figure 5B**). Interestingly there was a strong and significant interaction (*P* < 0.001) between cultivars, light intensity and inoculation indicating that not all genotypes responded in the same way. Thus, whereas under the normal light intensity the increases of night-time g_1_ were approximately of 131.7 and 82.7% for Cory and Orblanche, under the increased light intensity the night-time g_1_ increased, respectively, up to 203.1 and 110.8% with respect to non-inoculated plants. In addition Charming, that previously did not increase night-time g_1_, showed a slight stomatal lock-up when superimposing pathogen inoculation and higher light intensity. Overall, P01 barley did not respond to the increased light intensity with further nocturnal g_1_ increases, although it indeed increased g_1_ at the later time points of the time course (Supplementary Figure S1).

**FIGURE 6:**
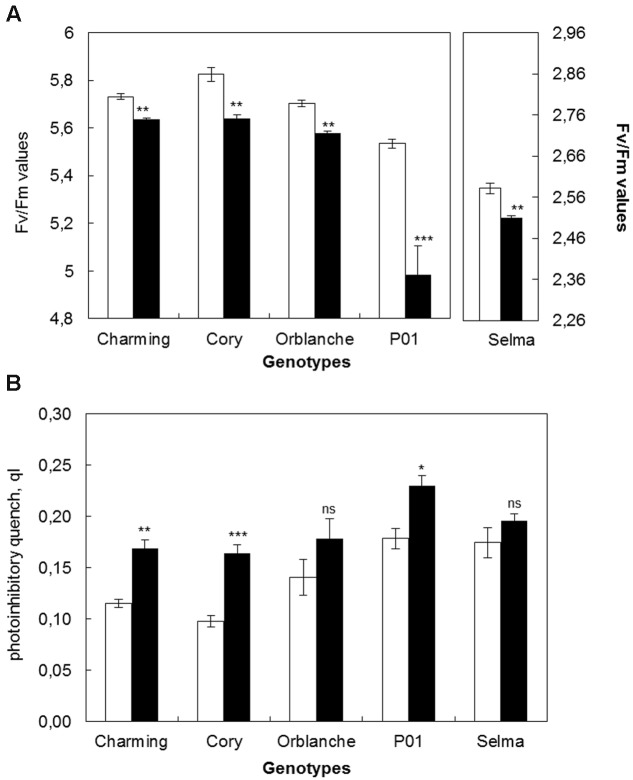
**Photosynthetic responses of oat and barley plants to powdery mildew attack and increased light intensity. (A)** Area Under the Conductance Progress Curve (AUPC) corresponding to a time course of Maximum Quantum Yield (Fv/Fm) measurements and **(B)** qI of healthy (white bars) Charming, Cory, Orblanche, and P01 leaves and inoculated plants (black bars). Plants were grown under a 12 h dark/12 h light cycles and a light regime of 250 μmol m^-2^s^-1^ and then transferred to a higher light intensity regime of 450 μmol m^-2^s^-1^ after inoculation. Bars represent the mean of 5 biological replicates ± standard errors. ^∗^, ^∗∗^, and ^∗∗∗^ indicate significant differences at *P* < 0.05, *P* < 0.01, and *P* < 0.001 respectively; ns indicates non-significant differences.

Regarding the effect of the moderate light increase in chlorophyll fluorescence parameters, there was an overall slight but significant (*P* < 0.001) decrease in the Fv/Fm values of the area under the progress curve from 5.73 to 5.58 (**Figure 6A**) but no significant effect on photoinhibition (**Figure 6B**). Confirming the previous results, under the moderate high light, pathogen challenge reduced significantly (*P* < 0.001) Fv/Fm ratios in all cultivars tested. However, there was no significant interaction between inoculation and light intensity, indicating that the overlapping of the increased light with the pathogen challenge did not change further the proportion of the Fv/Fm reduction previously observed. However, there were interactions between cultivars and inoculation and between cultivars and light intensity treatment. Thus, for instance, the resistant cv. Charming, that under the normal light assayed did not show photoinhibition following pathogen attack, showed it under the higher light intensity (**Figures 2B** and **6B**).

### Correlations between H_2_O_2_ Content, Stomatal Responses and Photosynthetic Electron Flow

**FIGURE 7:**
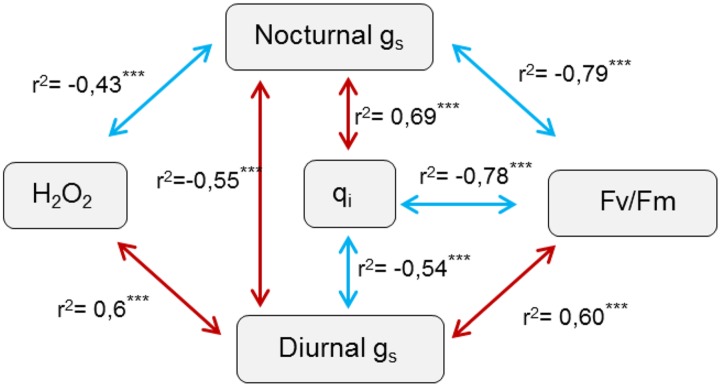
**Scheme of significant statistical correlations between physiological assessed parameters and hydrogen peroxide production.** Spearman correlations carried out based on the previously assessed physiological and H_2_O_2_ data of resistant oat and barley plants following pathogen attack. *r*^2^ indicate the correlation coefficient, with red arrows highlighting positives and blue arrows negative correlations respectively. ^∗∗∗^ indicate significant differences at *P* < 0.001 respectively.

**Figure 7** shows a scheme of statistically significant correlations found between H_2_O_2_ content, stomatal responses and chlorophyll fluorescence parameters assessed. It showed a significant correlation between H_2_O_2_ content and g_1_, this correlation being negative with night-time conductance but positive with daytime conductance. This data suggest that lower H_2_O_2_ content would favor high night-time and low daytime conductance as observed. There was no direct correlation between H_2_O_2_ content and chlorophyll fluorescence parameters. However, there was a strong correlation between stomatal conductance and photosynthetic electron flow estimates. Thus, night-time g_1_ was negatively correlated with Fv/Fm values, with a *r*^2^ coefficient of c.a 0.8, and positively correlated with q_i_. This suggested that high night-time g_1_ strongly influenced a reduction in Fv/Fm ratio and increased photoinhibition. On the other hand, daytime g_1_ followed an inverse trend suggesting that lower daytime g_1_ also affected negatively Fv/Fm values and increased photoinhibition. As expected Fv/Fm values were strongly and negatively correlated with q_i_ values. Interestingly, when taking into consideration only controls plants growing under the different light intensities a slight but significant positive correlation was observed between daytime and night-time g_s_ (*r*^2^ = 0.3, *P* < 0.01). However, when overlapping the inoculation treatment correlation turned to be negative (*r*^2^ = -0.55, *P* < 0.001).

## Discussion

One of the most important stomatal dysfunctions observed in the resistant oat cvs to powdery mildew was the loss of stomatal ability to close in darkness or lock-up. Since opening and close of stomata provide the necessary balance to maintain the relative concentration of CO_2_ for photosynthesis while preventing water losses (Roelfsema and Hedrich, 2005), one expected consequence of this altered stomatal behavior would be an alteration of the electron transport fluxes and ultimately of the carbohydrate balance. In line with this, we also observed an extensive alteration of chlorophyll fluorescence parameters such as the maximum quantum yield (Fv/Fm) during the execution of the resistance responses to *Bga*. This ratio has been widely used to detect stress-induced perturbations in the photosynthetic apparatus since decreases in this ratio reflect slowly relaxing quenching processes and photodamage of PSII reaction centers (Baker and Rosenqvist, 2004).

Stomatal lock-up have been related to the onset of HR in cucumber cotyledons infiltrated with avirulent bacteria (Pike and Novacky, 1988), and in a band of living epidermis surrounding necrotic lesions in potato leaf tissues killed by *Phytophthora infestans* (Farrell et al., 1969). Previously, we have shown lock-up in barley P01, P02, and P23 plants carrying the *Mla, Mla3 and MlLa1* genes respectively conditioning HR to the corresponding powdery mildew isolate (Prats et al., 2006, 2010; Mur et al., 2013). Thus, evidence from several different pathosystems indicates that death of cells is associated with stomatal dysfunctions. However, our current results suggest that there need not by a direct association between the extent of the HR and the derived stomatal alterations. Thus, cvs such as Cory, Orblanche and Charming exhibited a similar percentage of HR forming cells but differed in stomatal effects in response to *Bga.* This is of considerable practical importance since this offers the possibility of breeding for resistance either without, or with reduced, impact on stomatal dysfunction. Our previous work on barley genotypes, P01, P02, and P23, differing in the timing or localisation of HR but with similar HR percentages, showed similar stomatal dysfunction, in term of load, following powdery mildew challenge (Prats et al., 2010). Crucially, however, these genotypes are isogenic lines with a similar genetic background so that this, rather than *R* genes *per se*, would appear to be a major factor in conferring pathogen-responsive stomatal dysfunction. This suggestion is supported by some of our previous results in which stomatal dysfunction affected the barley genotype P22 but not Risø R, both recessive in the *mlo5 gene* but with different genetic background (Prats et al., 2006). In our current oat-based study, the different cultivars used also have different genetic backgrounds, arising from different breeding programs not sharing similar parents (Montilla-Bascón et al., 2013). Thus, data suggest that the stomatal effects are not ultimately dependent only in the percentage of dead cells, and water balance effects, but also in other processes triggered during both pre-penetration and post-penetration resistance. Both, pre-penetration and post-penetration resistance responses are highly complex and they are orchestrated by multiple genes and qualitative responses, so multiple genes present in the genetic background more than particular genes govern the final result (Bennet, 1984). For instance papilla-based penetration resistance involve several events including complex signal transduction (involving pH and Ca^+2^ changes, NO, H_2_O_2_ and other signaling molecules generation), formation of lipids microdomains, plasma membrane-cell wall adhesion, reorganization of the cytoskeleton, polarization of the cytoplasm and the endomembrane system, activation of transcription for synthesis of antifungal peptides, for secondary metabolites and for inhibitors of cell wall-degrading enzymes, synthesis of phytoalexins and monolignols, secretion of callose and other papilla components, phenolic and protein cross-linking, accumulation of H_2_O_2_ and other antifungal compounds (Hückelhoven, 2007).

In particular, it is known than the accumulation of reactive oxygen species (ROS) such as hydrogen peroxide or superoxide anion is one of the first responses of a plant to pathogen attack (Lamb and Dixon, 1997; Hückelhoven et al., 1999; Piffanelli et al., 2004). The ROS generated are not only direct protective agents, but also functions as a substrate for oxidative cross-linking in the cell wall, as a threshold for triggering hypersensitive cell death, and as a diffusible signal for induction of cellular protectant genes in surrounding cells. Thus, ROS generation is not only involved in HR development but in an overall resistance response including the papilla-based penetration resistance. In addition, a link between H_2_O_2_ and stomatal closure has also been well established (Pei et al., 2000). Using the fluorescent probe dichlorofluorescein it has been shown that generation of H_2_O_2_ is dependent on ABA concentration and that the H_2_O_2_ is required to initiate stomatal closure. Furthermore, when H_2_O_2_ is generated by an oligaracturonide elicitor or chitosan, stomatal closure occurs in tomato leaves (Lee et al., 1999). Indeed, we have observed closure of stomata at time of the oxidative burst in powdery mildew challenged resistant barleys (Prats et al., 2006, 2010). This led us to assess the levels of H_2_O_2_ for determining its possible involvement in the physiological dysfunctions observed later on after execution of the resistance responses. Interestingly, reduced levels of H_2_O_2_ were observed in inoculated respect to controls plants 2 and also as late as 5 d.a.i. when the direct resistance response should have been terminated. This reduction of the H_2_O_2_ levels might explain the failure of stomatal closure during the night-time, since as stated above, H_2_O_2_ is required for stomatal closure (Lee et al., 1999; Bright et al., 2006). The strong negative correlation found between H_2_O_2_ levels and night-time conductance support this hypothesis.

As expected, manipulation of H_2_O_2_ through increasing light intensity lead to overall elevated H_2_O_2_ levels both, in control and inoculated plants. However, it did not lead to direct cell damage, as indicated by CMS assessment. This suggests that observed effects on stomata might be more directly related to H_2_O_2_ signaling than with direct damage of the cell components. Interestingly, the H_2_O_2_ reduction of inoculated respect to control plants was still observed when overlapping pathogen inoculation under the higher light intensity. However, the proportional reduction of H_2_O_2_ in inoculated respect to controls slightly increased under the highest light intensity, Thus, not H_2_O_2_ levels *per se* but the proportional higher reduction respect to control levels might explain the higher lock-up observed when overlapping higher light intensity and pathogen inoculation. On the other hand, increased photosynthesis, which can be promoted by higher light intensity, has been reported to increase night-time stomatal openings (Easlon and Richards, 2009) and might lead to the stronger lock-up observed. In addition, the complex signaling cascade that orchestrates stomatal movement involves a myriad of other signals also involved in resistance responses, such as nitric oxide, polyamines, abscisic acid etc. so further work will be needed to dissect all these components and its relationship during the lock-up. The mechanisms by which H_2_O_2_ levels are reduced following effective resistance for relatively so long also need to be elucidated. It is known that at time of attempt penetration, *Blumeria graminis* up-regulate a gene encoding a catalase *Cat*B, which has been demonstrated to be secreted at the host-pathogen interface (Zhang et al., 2004). Furthermore, fungally derived scavenging of H_2_O_2_ was visualized around attack sites. However, we found genotype-dependent differences with Charming cv. showing fewer changes in H_2_O_2_ content and physiological dysfunctions than other resistant cvs. Thus, some of the mechanisms reducing the H_2_O_2_ are expected to relay on the host oxidant/antioxidant machinery.

Data showed no direct correlation between H_2_O_2_ levels and chlorophyll fluorescence parameters. However, chlorophyll fluorescence parameters were strongly correlated with both daytime and night-time conductance. This suggests that the disruption of photosynthetic electron transport is not a direct consequence of oxidative damage but it is a feature of pathogen/resistance-induced stomatal dysfunctions. It is worth mentioning that the resistant cv. Charming, similar to Cory or Orblanche in term of resistance, showed the lowest fluctuations on H_2_O_2_ levels after pathogen attack and also the lowest stomatal or photosynthetic dysfunctions. Although for several of the assessed parameters it could seem that Charming behave physiologically similarly to the susceptible cv. Selma following pathogen attack it is worth to note that, as stated previously, measurements in Selma finished earlier to avoid experimental interference with sporulation. Late effects of the powdery mildew disease development on susceptible cultivars involve impaired stomatal opening in the light and disease-induced senescence (reviewed by Ayres, 1981) but any of these effects are probably due to cell process and signaling different from those derived from the execution of the resistance mechanisms.

Although further work is needed to dissect the molecular bases governing the physiological dysfunctions derived from the resistance responses, our data, suggesting that background genotypic effects, involving a role for H_2_O_2_, rather than cell death *per se* are major determining factors, offer the possibility for such consequences to be avoided or reduced.

## Author Contributions

JS-M and GM-B carried out most of the experimental work and data analysis. DR and LM contributed to critical reading and writing and discussion of the results. EP designed experiments, and contributed to the interpretation and discussion of results and writing of the manuscript.

## Conflict of Interest Statement

The authors declare that the research was conducted in the absence of any commercial or financial relationships that could be construed as a potential conflict of interest.

## Abbreviations

AL: actinic light
*Bga*: *Blumeria graminis* f. sp. *avenae*
*Bgh*: *Blumeria graminis* f. sp. *hordei*
F_0_: initial fluorescence level
F_m_: maximal fluorescence value
F_m_′: maximum fluorescence level
F_q_’/F_m_: PSII operating efficiency (ΦPSII)
Fv/Fm: Maximum Quantum Yield of PSII
g_1_: leaf water conductance
h.a.i: hours after inoculation
PS: photosystem
q_I_: photoinhibitory quench
