# Supplementary material for: Compromised Photosynthetic Electron Flow and H2O2 Generation Correlate with Genotype-Specific Stomatal Dysfunctions during Resistance against Powdery Mildew in Oats
Source: Front Plant Sci. 2016 Nov 8;7:1660. doi: 10.3389/fpls.2016.01660 (PMC5099169; doi:10.3389/fpls.2016.01660)
Supplement: Supplementary file 1 [file Data_Sheet_1.PDF]

Supplementary Material

Compromised Photosynthetic Electron Flow And H2O2 Generation Influence Genotype-Specific Stomatal Dysfunctions During Resistance Against Powdery Mildew In Oats.

Javier Sánchez-Martín1, Gracia Montilla-Bascón, Luis AJ Mur2, Diego Rubiales1 and Elena Prats1*.

[1]Institute for Sustainable Agriculture-CSIC, Córdoba, Spain.
[2]Institute of Biological, Environmental and Rural Sciences, University of Aberystwyth, UK

*** Correspondence:** Dr. Elena Prats, Institute for Sustainable Agriculture, CSIC, Apdo. 4084, E-14080 Córdoba, Spain.
elena.prats@ias.csic.es

Supplementary Figure 1

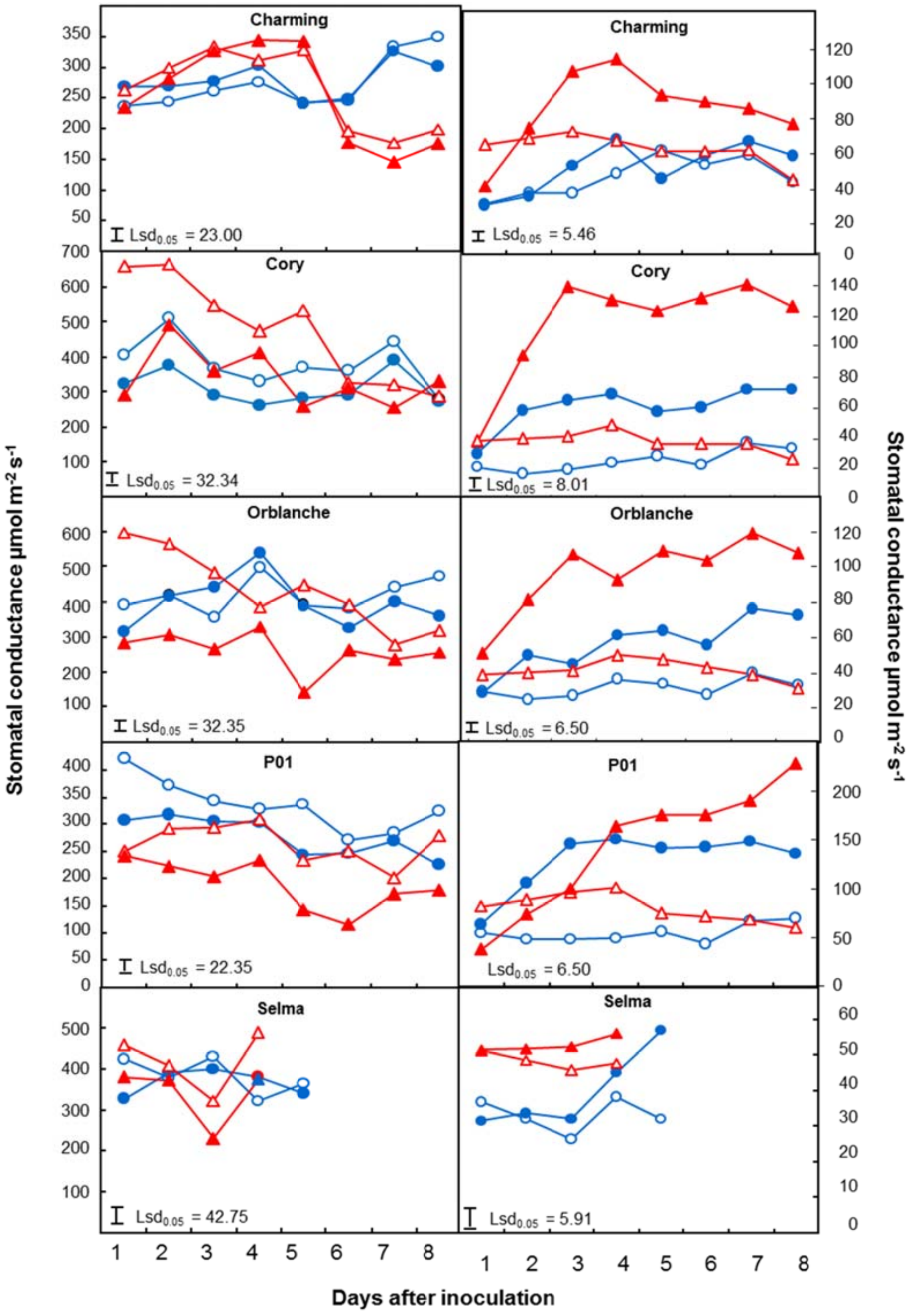

Supplemental Figure 1. Time course of A) diurnal and B) nocturnal leaf water conductance (g1) of healthy (open) Charming, Cory, Orblanche, P01 and Selma leaves and inoculated plants (solid) with Blumeria graminis f. sp. avenae in mid-light period, when incubated under a 12 h dark/ 12 h light cycles under two different light regimes, low light intensity (250 µmol m-2 s-1, circles, blue) and moderate high light intensity (450 µmol m-2 s-1, triangles, red). Segments indicate 95% confidence intervals. Bars represent the mean of 10 replicates
